# How or When Samples Are Collected Affects Measured Arsenic Concentration in New Drinking Water Wells

**DOI:** 10.1111/gwat.12643

**Published:** 2018-03-06

**Authors:** Melinda L. Erickson, Helen F. Malenda, Emily C. Berquist

**Affiliations:** ^1^ Colorado School of Mines 1500 Illinois St. Golden CO 80401; ^2^ Minnesota Department of Health 625 Robert Street North St. Paul MN 55155

## Abstract

Naturally occurring arsenic can adversely affect water quality in geologically diverse aquifers throughout the world. Chronic exposure to arsenic via drinking water is a human health concern due to risks for certain cancers, skin abnormalities, peripheral neuropathy, and other negative health effects. Statewide in Minnesota, USA, 11% of samples from new drinking water wells have arsenic concentrations exceeding 10 μg/L; in certain counties more than 35% of tested samples exceed 10 μg/L arsenic. Since 2008, Minnesota well code has required testing water from new wells for arsenic. Sample collection protocols are not specified in the well code, so among 180 well drillers there is variability in sampling methods, including sample collection point and sample collection timing. This study examines the effect of arsenic sample collection protocols on the variability of measured arsenic concentrations in water from new domestic water supply wells. Study wells were drilled between 2014 and 2016 in three regions of Minnesota that commonly have elevated arsenic concentrations in groundwater. Variability in measured arsenic concentration at a well was reduced when samples were (1) filtered, (2) collected from household plumbing instead of from the drill rig pump, or (3) collected several months after well construction (instead of within 4 weeks of well installation). Particulates and fine aquifer sediments entrained in groundwater samples, or other artifacts of drilling disturbance, can cause undesirable variability in measurements. Establishing regulatory protocols requiring sample filtration and/or collection from household plumbing could improve the reliability of information provided to well owners and to secondary data users.

## Abstract 2

Article impact statement: Water sampling protocols—filtration, timing, sample point—influence variability in arsenic concentrations in new drinking water wells.

## Introduction

Arsenic (As) is a naturally occurring trace element that can adversely affect drinking water quality sourced from groundwater in geologically diverse aquifers in Asia, Europe, Africa, and North and South America. In some cases, elevated aqueous As concentrations are confined to certain aquifer sediments because of specific geologic history combined with hydrological, geochemical, and biological conditions (Stuckey et al. 2015). Examples include the sulfide cement horizon in eastern Wisconsin, USA (Schreiber et al. 2000; Gotkowitz et al. 2004) or groundwater downgradient from a petroleum spill (Cozzarelli et al. 2016). However, areas of As mobilization from sediment to groundwater are found widely around the world via the reductive or oxidative mechanisms naturally present (Welch et al. 2000; Smedley and Kinniburgh 2002; Shankar et al. 2014). Chronic exposure to arsenic via drinking water is a human health concern due to risks for certain cancers, skin abnormalities, peripheral neuropathy, and other negative health effects (Mayer and Goldman 2016; US Environmental Protection Agency 2017). Arsenic concentration variability has been observed in some studies due to local flow‐system changes (Gotkowitz et al. 2004; Erickson and Barnes 2006; Ayotte et al. 2011; Bexfield and Jurgens 2014) or seasonal differences (Ayotte et al. 2015).

The US Environmental Protection Agency's (EPA) Maximum Contaminant Level (MCL) and the World Health Organization's guideline for drinking water arsenic concentration is 10 micrograms per liter (μg/L) (US Environmental Protection Agency 2017; World Health Organization 2017). The MCL is enforceable at public water systems but not at domestic (private) wells. EPA's MCL Goal (MCLG), a non‐enforceable public health goal, is 0 μg/L. For the purpose of this paper “elevated” is defined as ≥10 μg/L even though chronic exposure to arsenic at lower concentrations can be detrimental.

Naturally occurring elevated As concentration in groundwater is common throughout the upper Midwest, USA (Erickson and Barnes 2005; Warner and Ayotte 2014). In this region, As concentration is commonly elevated in glacial and shallow bedrock aquifers that lie within the area of the northwest provenance of Late Wisconsin‐aged glaciations (Des Moines Lobe; Erickson and Barnes 2005). The geology of Minnesota, USA, is similar to other states in the upper Midwest, and is composed of bedrock overlain by multiple layers of glacial sediment deposited by successive glaciations.

Since 2008, the Minnesota well construction code has required that samples from new water wells be tested for As concentration because of the frequency of elevated As concentration in Minnesota's drinking water aquifers (State of Minnesota 2017). From 2008 to 2017 samples from more than 43,000 new drinking water wells in Minnesota have been tested for As concentration, and summary results are available online (Minnesota Department of Health 2017c). In Minnesota, approximately 11% of all tested drinking water wells have elevated As concentrations, with some counties having samples from more than 35% of tested new wells with concentrations above the 10 μg/L MCL (Minnesota Department of Health 2017c). An estimated 130,000 domestic water well users in Minnesota are exposed to elevated drinking water As concentrations (Minnesota Department of Health 2017a). Samples from more than 47% of all tested Minnesota drinking water wells, serving approximately 550,000 people, have As concentrations above 2 μg/L, the typical commercial laboratory reporting level.

Water quality testing (well water samples are tested for nitrate and bacteria in addition to As concentration) at new drinking water wells is the responsibility of the approximately 180 licensed well construction contractors (well drillers). The well code does not specify a sampling point, and neither field‐filtration nor field‐acidification of the sample are required. Arsenic analyses must be done by one of the approximately 16 private laboratories certified by the State of Minnesota to analyze As concentration in drinking water. Laboratories provide sample containers and instructions for sample collection to well drillers. Drillers collect water for As analysis either using their drill rig pump immediately after well development (sample collection from the rig), or later through a completed drinking water system (sample collection from household plumbing).

Although required testing of samples from new wells is the responsibility of well drillers, private well owners are not required to comply with the 10 μg/L MCL set by EPA. Homeowners are provided with public health information about their water quality results, but they are responsible for any additional water quality testing or treatment of their well water. Water treatment systems can be expensive, so it is important for the homeowners to know the expected long‐term As concentration in their private well water before deciding on an appropriate water treatment option. This study examines the effects that different groundwater sampling protocols and sample collection timing have on measured As concentration in drinking water over a one‐year timespan. The results have relevance to public health and policy.

## Materials and Methods

### Study Area

**Figure 1:**
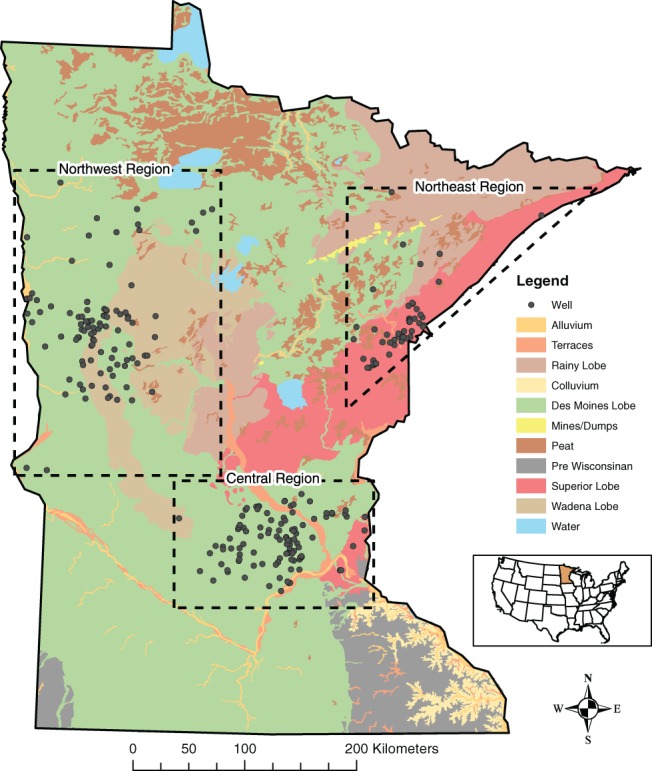
Study areas. Simplified surficial glacial geology (simplified from Hobbs and Goebel 1982), and sampled well locations.

This study focuses on three regions of Minnesota that differ geologically: Northeast, Northwest, and South‐central (hereinafter called Central) (Figure 1). These areas were chosen due to their frequently measured elevated As concentrations in drinking water. These regions have common drinking water aquifer characteristics (disconnected or spatially extensive sand and gravel aquifers interbedded with aquitards; or bedrock) reflected around the globe. Little is known about the mechanism of arsenic occurrence in bedrock wells in the Northeast region. Mechanisms for arsenic release to groundwater in the Northwest and Central regions (reductive desorption of As(V), and reductive dissolution of iron (oxyhydr)oxides) have been described in Erickson and Barnes (2005), and in the Northwest region [incongruent dissolution, or rapid oxidation of iron sulfides and re‐precipitation of Fe as (oxyhydr)oxides] by Nicholas et al. (2017). This study examines the question of whether arsenic concentrations systematically decrease over time in well water following potable domestic well installation and completion in Minnesota. We are unaware of any studies that addresses this question.

#### Northeast

The Northeast study region is geologically composed of crystalline bedrock overlain by glacial till deposited by the Rainy Lobe and by the Superior Lobe from the northeast Labrador ice center in Canada (Hobbs and Goebel 1982; Lusardi 1997; Meyer 1997; Knaeble and Hobbs 2009). In the Northeast region, drinking water wells are drilled in glacial aquifers or crystalline bedrock aquifers. Precambrian crystalline bedrock predominantly underlies the glacial till, and bedrock aquifers provide drinking water to a high percentage of domestic and municipal wells. Glacial sediment in this region has a high percentage of sand and pebbles with a reddish‐brown to reddish‐gray color (Knaeble and Hobbs 2009). Glacial aquifers within the Superior Lobe till are composed of pockets of sand and gravel that typically report no or very low measured groundwater As concentrations. In this region, As is primarily found in groundwater from bedrock wells. Although water samples from most bedrock wells have As concentration < 10 μg/L, the uncommon As concentration detections above 10 μg/L can be 100 μg/L or more (Minnesota Department of Health 2016b). About half of the study wells in this region were bedrock wells, and about half were glacial wells screened in sand and gravel.

#### Northwest

The Northwest study region is composed of complex and heterogeneous sediments deposited from and distributed by many glacial advances and retreats, as well as sediments deposited in glacial lakes. Aquifers used for drinking water in the Northwest study region tap glacial sand and gravel deposits of varying depths and thicknesses, with drinking water well depths ranging from 5.5 to 145.5 m (Minnesota Department of Health 2017b). Shallow sand and gravel aquifers (<30 m deep) within moraine and outwash landforms provide drinking water to domestic wells (Ekman and Berg 2002). These aquifers are surrounded by till that is unsorted clay‐rich glacial material ranging in grain size from clay to boulders. This till is rich in shale and limestone clasts. Elevated arsenic concentrations occur most frequently in this region. Lacustrine sediments of silt and clay were deposited in many small glacial lakes throughout the region and over a large part of the region in Glacial Lake Agassiz (Clayton and Moran 1982; Gowen 2014; Gowan and Marshall 2016). All of the study wells in this region were screened in glacial sand and gravel.

#### Central

Like the Northwest region, the Central region is composed of a complex distribution and layering of glacial sediment, but this region experienced fewer glacial advances and less layering than the Northwest region (Lusardi et al. 2011). Similar to the Northwest region, drinking water in the Central region comes from sand and gravel aquifers embedded in glacial till. Till layers laterally extend over large areas, with meltwater‐deposited sand and gravel at lower drainage elevations (Lusardi and Jennings 2009; Lusardi and Lively 2009; Knaeble 2013). The clay‐rich till forms confining layers over the aquifers and contributes to a frequency of elevated arsenic concentrations in groundwater that is about double the statewide average (Minnesota Department of Health 2017c). The majority of glacial sediment deposited by meltwater consists of poorly sorted pebbles, cobbles, limestone fragments, clay, sand, and gravel (Clayton and Moran 1982; Lusardi and Jennings 2009; Lusardi and Lively 2009). Erosion and deposition by advancing and retreating glaciers created pockets of coarse‐grained material that forms aquifers used for water supply. Drinking water wells in the Central region are drilled in both glacial aquifers and sedimentary bedrock aquifers. Glacial aquifers support a large number of domestic wells, while bedrock aquifers supply primarily higher‐capacity municipal drinking water (Lusardi and Jennings 2009; Lusardi and Lively 2009; Petersen 2013). Most (about 95%) of the study wells in this region were screened in glacial sand and gravel.

### Groundwater Sample Collection and Laboratory Analysis

**Figure 2:**
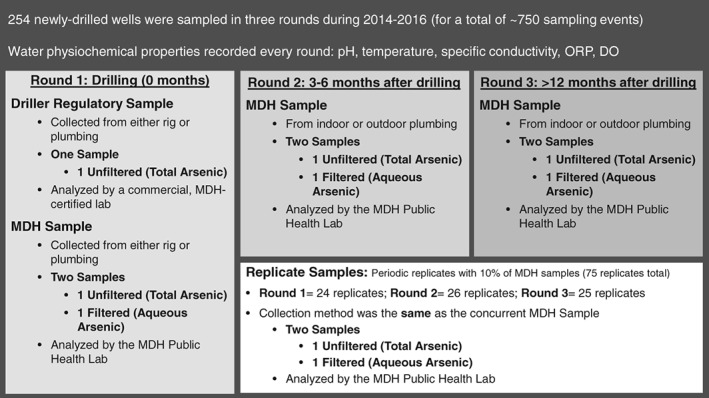
Study design. Illustration of sample collection protocols, timing, laboratory, and replicate sample collection over the study. ORP, oxidation–reduction potential; DO, dissolved oxygen.

From 2014 to 2016, approximately 250 new private domestic water wells were sampled by MDH staff in three rounds (for a total of ∼750 sampling events). The timing of the three sampling rounds was (1) within 4 weeks of well construction (Round 1); (2) 3–6 months after initial sample collection (Round 2); and (3) >12 months after initial sample collection (Round 3). Each sampling event included collection of both filtered and unfiltered groundwater samples, as well as field measurement of physicochemical properties (specific conductance, pH, dissolved oxygen [DO], redox potential [ORP], and water temperature) (Figure 2) to determine stability.

The initial sampling event was timed to co‐occur and mimic regulatory sampling for total As concentration (State of Minnesota 2017). Regulatory water sample collected by the drilling contractors are not filtered or field‐preserved with acid. Rather, whole‐water samples were collected and sent to one of the various MDH‐certified laboratories for total As concentration analysis according to procedures established by the laboratory. Regulatory samples were collected either from the drill rig after well development using the drill rig groundwater pump, or later from the household plumbing. The maximum laboratory reporting level for mandatory regulatory samples was 2 μg/L As, which is a requirement for state certification and ensures that homeowners are informed of all arsenic concentrations greater than 2 μg/L.

Initial water samples collected by MDH staff were taken directly from the drill rig groundwater pump or from the household plumbing, replicating the method used by the well driller. Sampling from the drill rig's groundwater pump occurred after the well was drilled and developed, when the water was visibly clear, with little visible sediment particles. Samples from plumbing were collected after the plumbing was flushed out and field measurements of physicochemical properties stabilized. The second and third sampling rounds by MDH staff were collected only from plumbing. Samples collected from plumbing were taken from indoor or outdoor faucets, or from pressure tanks prior to filters or treatment systems.

All sampling performed by MDH staff followed the method protocol for total As and aqueous As (Table S1, Supporting Information). Groundwater samples for aqueous As analysis were filtered in‐field through a 0.45 μm filter. All MDH groundwater samples for As analysis were field‐preserved with metal‐free nitric acid and immediately put in coolers with ice or refrigerated at 4 °C until delivery to the laboratory. Groundwater samples were analyzed at the MDH Public Health Laboratory (MDH PHL) in St. Paul, Minnesota. Total As and aqueous As were analyzed using the standard methods of the U.S. Environmental Protection Agency (EPA) 200.8 (US Environmental Protection Agency 1994). The laboratory reporting level for the MDH PHL was 1 μg/L As.

### Dataset Details and Quality Assurance

In the Northeastern, Northwestern, and Central regions 49, 101, and 104 wells were sampled, respectively. The study design is described in Figure 2. Replicate samples were collected for 10% of the MDH samples (75 replicates total). Replicate sampling occurred periodically (at approximately every 10th well) during the three rounds and across the three regions. Replicates were collected at the same time and in the same manner as their respective reference MDH Lab samples. For detectable total and aqueous As, the average relative percent differences (RPDs) among the 75 pairs were 4% and 3%, respectively. The maximum RPD was an outlier at 28% (Table S2). Additionally, Paired Prentice‐Wilcoxon tests (PPW test; see Statistical Methods, below) indicated that distributions of reference and replicate determinations were similar for both total As (p = 0.28) and aqueous As (p = 0.13) (Table S3). During the sampling campaign, 10 sample blanks were collected using metal‐free water provided by the MDH PHL. No target analytes were detected in any of the blank samples (Table S4).

### Statistical Methods

To compare analyte concentrations across time and sampling methods for the study wells, we used the nonparametric PPW test (O'Brien and Fleming 1987). Unlike summary statistics, which compare dataset distributions, pair‐wise comparisons offer additional information regarding changes across wells and their rank in their respective datasets. This pairwise method can be used on datasets that are heavily‐censored (Cuzick 1982; O'Brien and Fleming 1987), and both normally and non‐normally distributed (O'Brien and Fleming 1987). The PPW test was also chosen for this study because unlike conventional signed‐rank tests, the PPW test is sensitive to the degree of difference between paired data points' positions in their respective distributions (O'Brien and Fleming 1987). Additionally, the PPW method's null hypothesis of similarity between the positions of matched‐pairs' data points also requires the two distributions' similitude.

In the PPW method, the two datasets being compared are combined, and a Kaplan–Meier survival function and associated scores are estimated for every data point in the commingled dataset (Helsel 2012). The data sets are then divided into their original arrays. The differences between scores for each pair, as well as a subsequent test statistic (Z‐score) for the paired distributions, are calculated. The more similarity between paired data points' positions in their respective distributions, the smaller the differences between pairs' scores, the smaller the distributions' Z‐score, and the greater the resulting p‐values (O'Brien and Fleming 1987; Helsel 2012).

Additionally, the PPW test is well‐suited to handle censored data with reporting limits that vary within distributions, as long as the reporting limits of a matched‐pair are the same. Reporting limits that consistently differ between the two groups will create bias in the PPW method toward dissimilarity (Helsel 2012). To prevent this bias, both distributions can be recensored to the higher reporting limit (O'Brien and Fleming 1987; Helsel 2012). Helsel (2012) also suggests common recensoring because it eliminates interblock information bias (O'Brien and Fleming 1987). In this study, arsenic concentrations were recensored to the higher reporting limit of 2 μg/L for PPW tests comparing driller and MDH datasets. For PPW tests comparing MDH analyte data between the three sampling rounds, no recensoring was required.

**Figure 3:**
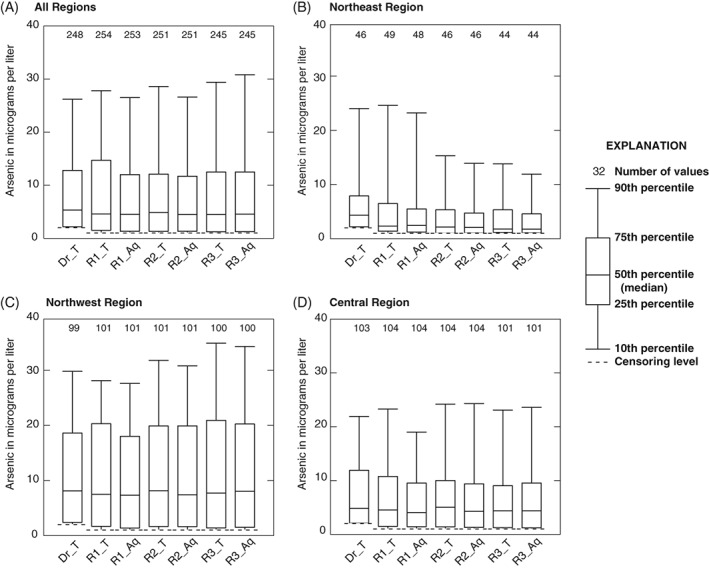
Distribution of arsenic concentrations, by region over time (Dr, driller‐collected; T, total arsenic; Aq, aqueous arsenic; R1, round 1 Minnesota Department of Health [MDH] sample; R2, round 2 MDH sample; R3, round 3 MDH sample). Box plots illustrate no notable change in total or aqueous arsenic concentrations over time within regions.

## Results

**Figure 4:**
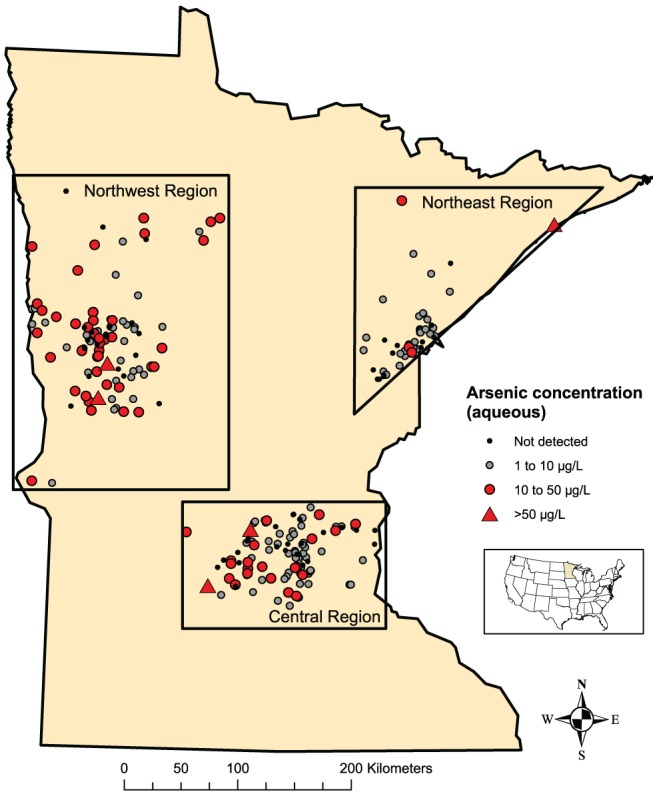
Arsenic concentrations in well water. Concentrations of arsenic in newly constructed wells approximately 1 year after well drilling. Aqueous arsenic concentrations from Round 3 depicted. μg/L, micrograms per liter.

Figure 4 shows a map of Round 3 aqueous As concentrations. Figure 3 shows summary statistics for the arsenic samples in the form of box plots. Box plots show all measured As concentrations during the three study rounds, both as a whole, and by geographic region. Summary statistics documenting the amount of time (days/months) between Round 1 and Rounds 2 and 3 are provided in the Supplemental Information (Table S5). The full data set is available online from the USGS ScienceBase Catalog (Krall et al. 2018).

### Groundwater Chemistry

General summary statistics as represented by box plots can be used to compare the distribution of As concentrations between regions and across sampling rounds. Figure 3 indicates that total and aqueous As concentrations in new wells, as measured by summary statistics, are similar over time during the study. The number of aqueous As concentration measurements that exceeded 10 μg/L was similar over the three sampling rounds, with approximately 28% of study As concentrations being greater than 10 μg/L. The Northeast region had the lowest median (1.76 to 4.43 μg/L, combined total and aqueous As concentrations over the three rounds), with approximately 10% of samples having elevated aqueous As concentrations. The Northwest region had the highest median (7.31 to 8.13 μg/L) As concentrations, and the largest number of elevated As concentration measurements, with approximately 40% of aqueous As concentrations greater than 10 μg/L in each sampling round. The Central region had median As concentrations from 4.05 to 5.05 μg/L, with approximately 25% of aqueous As concentrations greater than 10 μg/L.

### Variability in Measured Arsenic Concentrations

Matched PPW results reveal important trends in dissimilarity of measured As concentrations at individual wells, comparing sampling protocol differences of filtration, sample collection point, and sample collection timing. These dissimilarities cannot be detected by evaluating summary statistics.

The PPW test was applied to composite datasets that included data from wells in all three regions. The driller‐collected total As concentration dataset is dissimilar to all MDH‐collected As datasets (both total and aqueous), in all three sampling rounds (Table 1, top; Table S6). Similarly, the Round 1 (0‐month) MDH‐collected total As concentration data are also dissimilar to all other As concentration datasets, both total and aqueous, across all three sampling rounds. The initial MDH aqueous As concentration dataset, however, is not different from the remaining MDH As concentration datasets from the second and third sampling rounds (Table 1, top). Additionally, five out of six pairs of datasets from the second and third sampling rounds are not different from one another. Except for the initial driller‐ and MDH‐ collected total As concentration datasets, both aqueous and total As concentration datasets from the Round 3 (>12‐month) round are similar to all other datasets. These results indicate that initial measured total arsenic concentrations (unfiltered samples collected near to the time of well drilling) are different from filtered or later arsenic measurements.

**Table 1:** Paired Prentice‐Wilcoxon Test Results

Paired Prentice‐Wilcoxon Test Results		Driller	MDH Collection				
		0 Months. TAs	0 Months. TAs	0 Months. AqAs	3–6 Months. TAs	3–6 Months. AqAs	12 Months. TAs
**All regions**							
**MDH Collection**	**0 months. TAs**	Different	—	—	—	—	—
	**0 Months AqAs**	Different	Different	—	—	—	—
	**3–6 Months TAs**	Different	Different	**Not different**	—	—	—
	**3–6 Months AqAs**	Different	Different	**Not different**	Different	—	—
	**12 Months TAs**	Different	Different	**Not different**	**Not different**	**Not different**	—
	**12 Months AqAs**	Different	Different	**Not different**	**Not different**	**Not different**	**Not different**
**Central**							
**MDH Collection**	**0 Months TAs**	**Not different**	—	—	—	—	—
	**0 Months AqAs**	Different	Different	—	—	—	—
	**3–6 Months TAs**	**Not different**	**Not different**	**Not different**	—	—	—
	**3–6 Months AqAs**	Different	Different	**Not different**	Different	—	—
	**12 Months TAs**	Different	**Not different**	**Not different**	**Not different**	**Not different**	—
	**12 Months AqAs**	Different	**Not different**	**Not different**	**Not different**	**Not different**	**Not different**
**Northwest**							
**MDH Collection**	**0 Months TAs**	**Not different**	—	—	—	—	—
	**0 Months AqAs**	**Not different**	Different	—	—	—	—
	**3–6 Months TAs**	**Not different**	**Not different**	Different	—	—	—
	**3–6 Months AqAs**	**Not different**	**Not different**	Different	**Not different**	—	—
	**12 Months TAs**	**Not different**	**Not different**	Different	**Not different**	**Not different**	—
	**12 Months AqAs**	**Not different**	**Not different**	Different	**Not different**	**Not different**	**Not different**
**Northeast**							
**MDH Collection**	**0 Months TAs**	**Not different**	—	—	—	—	—
	**0 Months AqAs**	Different	**Not different**	—	—	—	—
	**3–6 Months TAs**	Different	Different	**Not different**	—	—	—
	**3–6 Months AqAs**	Different	Different	Different	**Not different**	—	—
	**12 Months TAs**	Different	**Not different**	**Not different**	**Not different**	**Not different**	—
	**12 Months AqAs**	Different	**Not different**	**Not different**	**Not different**	**Not different**	**Not different**

Notes: Test result p‐values <0.05 (Table S6) reject the null hypothesis that the distributions are the same. AqAs, aqueous arsenic; MDH, Minnesota Department of Health; Mo, month; TAs, total arsenic.

**Figure 5:**
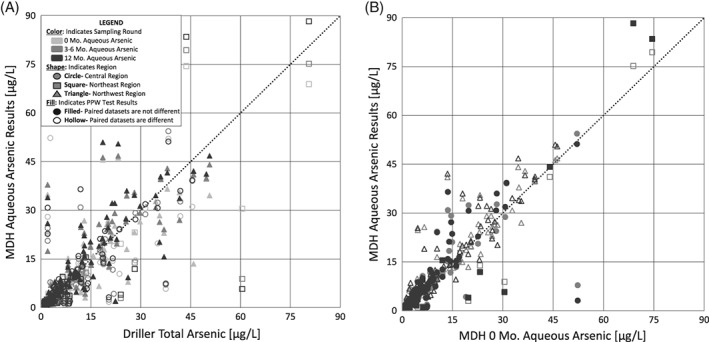
Paired Prentice‐Wilcoxon (PPW) test results reveal differences in total and aqueous arsenic concentration over time. Pair‐wise comparison of samples illustrate that (a) initial total (unfiltered) samples are more different (hollow symbols) from later samples, while (b) initial aqueous (filtered) samples are more similar (filled symbols) to later samples. Mo., month; MDH, Minnesota Department of Health; μg/L, micrograms per liter.

When the PPW test is applied to regional subsets of the whole data set, patterns different from the whole data set analysis emerge (Figure 5, Table 1, Table S6). For example, in the Northwest region both the driller‐ and initial MDH‐collected total As concentration datasets are similar to all other As concentration datasets, while the initial MDH‐collected aqueous As concentration dataset is different from all other As concentration datasets. These three results contrast those of the central and northeast regions (Figure 5, Table 1; Table S6). Additionally, except for the comparison of the 3–6 month total and 3–6 month aqueous As concentration datasets in the Central region, all second (3–6 month) and third (>12‐month) round As concentration datasets are not different from each other.

In the Central region we applied PPW tests to compare the two typical driller sample‐collection points: from the drill rig pump or from plumbing. The driller‐collected total As concentration data were broken into Round 1 subsets by sampling points (from rig or from plumbing). These subsets of driller‐collected sample results were compared to MDH data subsets from Rounds 1, 2, and 3 (Table 1). As discussed in the methods, Round 1 (0‐month) water samples collected by MDH staff were taken directly from the drill rig groundwater pump or from the household plumbing, replicating the method used by the well driller. The two driller data subsets were compared to the two sampling method data subsets collected by MDH during the first sampling round (i.e., Driller rig vs. MDH rig subsets and Driller plumbing vs. MDH plumbing subsets). Because we employed the PPW statistical test, the MDH data subsets used in each comparison only included those wells which were included in the respective driller‐collected data subset. The two driller data subsets (rig and plumbing) were then compared to their respective Round 2 and 3 data subsets. The rig vs. plumbing comparison was not performed in the Northeast and Northwest regions due to the small number of rig sampling points.

The PPW test results indicate that when collected from plumbing, unfiltered driller‐collected As concentrations are not different from total or aqueous As concentrations over the course of a year (all three rounds collected by MDH, Table 2). In contrast, driller‐collected samples from the rig are different from all aqueous As samples (Rounds 1–3) and all later total samples (Rounds 2 and 3). As expected, the driller‐collected samples from the rig are similar to the MDH‐collected total As samples collected from the rig, but are different from all of the other samples.

**Table 2:** Paired Prentice‐Wilcoxon Test Results for Samples Collected from a Drill Rig vs. Samples Collected from Plumbing

Driller v MDH Common Recensoring	MDH Collection							
	0 Months Plumbing		0 Months Rig		3–6 Months Plumbing		12 Months Plumbing	
Driller Collection	TAs	AqAs	TAs	AqAs	TAs	AqAs	TAs	AqAs
0 Months Plumbing	**Not different**	**Not different**	—	—	**Not different**	**Not different**	**Not different**	**Not different**
0 Months Rig	—	—	**Not different**	Different	Different	Different	Different	Different

AqAs, aqueous arsenic; MDH, Minnesota Department of Health; TAs, total arsenic.

**Figure 6:**
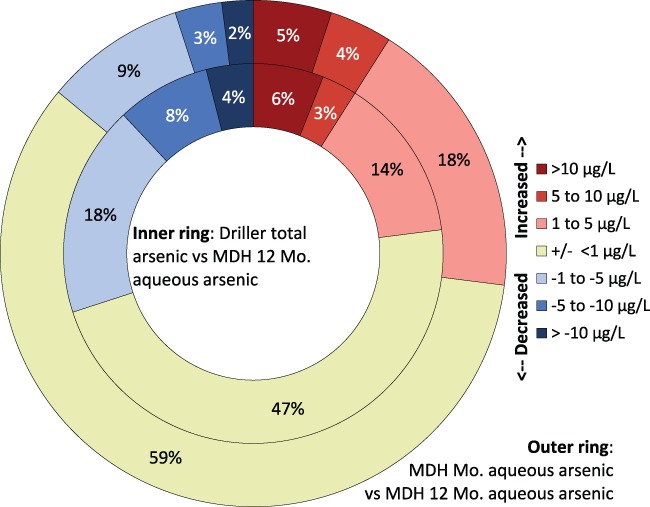
Magnitude and direction of arsenic concentration change over time. Later measured arsenic concentrations fluctuate both up and down, compared to initial measured arsenic concentrations. The percentage of wells with large arsenic concentration increases (>10 μg/L increase) is higher than the percentage of wells with large arsenic concentration decreases. MDH, Minnesota Department of Health; Mo., month; μg/L, micrograms per liter.

Figure 6 illustrates the magnitude and direction (increase or decrease) of differences in As concentration over a year between driller‐collected (unfiltered) As samples and field‐filtered samples collected alongside the driller sample. Initial (Round 1) samples are compared to the >12‐month (Round 3) aqueous samples. A larger percentage of sample‐pairs have only small concentration differences over time when samples are field‐filtered than when samples are not filtered. Conversely, a larger percentage of sample‐pairs have large differences (more than 10 μg/L change in concentration) in the unfiltered samples when compared to Round 3 field‐filtered samples.

## Discussion

### Effect of Sample Filtration, Sample Collection Point, and Sample Collection Timing on Arsenic Concentration

This study demonstrates that several aspects of groundwater sampling protocols affect As concentrations present in well water samples.

#### Sample Filtration

Field‐filtering samples from new wells yields less variability of measured As concentration over time when compared to unfiltered samples. Although it is generally desirable to collect unfiltered drinking water samples, collection of filtered arsenic samples right after well drilling may be desirable. Drill rig pumps do not replicate or reflect domestic well pump operations. Drill rig pumps run at a very high flow rate and are purposefully used to induce high velocities at the well screen to remove fine particles from the boring and gravel pack. Typical domestic well pump operation results in lower flow rates with less disturbance or entrainment of aquifer materials. As the study shows, filtration of samples collected shortly after well construction reduces reported As concentration variability as quantified in two different ways.

**Figure 7:**
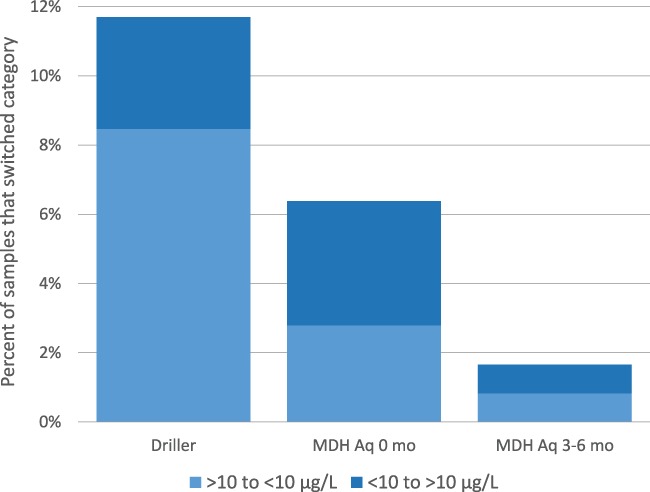
Summary of the percent of wells that change As concentration category (from above to below the threshold of 10 μg/L, or vice versa) over time. Comparison is between the indicated sample to the Round 3 (12‐month) aqueous arsenic sample. MDH, Minnesota Department of Health; Aq, aqueous arsenic sample; mo, month; μg/L, micrograms per liter.

Figure 7 illustrates the percentage of wells that changed As concentration category (from above to below the 10 μg/L or vice‐versa) over time. Simply filtering field samples reduced the number of sample locations that changed from above to below, or from below to above, 10 μg/L. Regulatory driller‐collected, unfiltered samples, compared to aqueous samples collected during Round 3, switched between As categories in almost 12% of samples. Filtering initial samples reduced category changes by about 50% (to 6% of samples).

Filtering samples also reduces the magnitude of concentration changes (Figure 6). Although large changes in As concentration do not necessarily result in a change in As category (above or below 10 μg/L), large changes in As concentrations can nonetheless be disconcerting and confusing to well owners and can have health implications. EPA's MCLG is 0 μg/L As concentration in drinking water, and current advice from MDH to well owners is to consider treating drinking water if As is detected at any concentration.

Filtration of samples reduces the variability of measured As concentrations over time because solid material entrained in water in sample bottles can interact with As, which affects its measured concentration. Filtering removes entrained sediment. If the entrained solids are high in carbonate (as they sometimes are in Minnesota water samples), then the solids can negate the acid preservative. Inadequate acidification allows aqueous As to adsorb to solids in the sample bottle (Pierce and Moore 1982; De Vitre et al. 1991; Welch et al. 2000; Bose and Sharma 2002; Sracek et al. 2004; Erickson 2005). This type of interaction will bias the measured As concentration low. This low measurement bias could pose a risk to public health because homeowners may erroneously receive As concentration results that indicate there is no or little risk to them from As in their new water supply well.

In contrast, if the entrained solids are high in iron oxides or sulfides and acid preservative is added to the sample bottle, As that had originally been sorbed to iron oxides or bound within the matrix of iron sulfides can be mobilized into the aqueous phase (Bose and Sharma 2002; Erickson 2005). This type of interaction will bias measured As concentrations high. A negative consequence of measured As concentrations being higher than the actual As concentration is a potential increased cost to homeowners who choose to install potentially unnecessary or oversized drinking water treatment systems.

Field‐filtering protocols for samples collected for As analysis to comply with well construction code for potable water supply could be communicated to well‐drilling contactors, homeowners, and others, and could be implemented. All parties could benefit from consistent sample collection protocols.

#### Sample Collection Point

Collection of samples from plumbing rather than from the drill rig also reduces variability of measured As concentration over time. Initial total As samples taken from plumbing were not different from either aqueous or total As concentrations collected from plumbing over the course of a year following well construction (Table 2). This was not the case with unfiltered samples collected from the rig. The subset of total As concentration samples collected from the rig was dissimilar to *all* samples collected from plumbing. It is reasonable to expect that less solid material will be entrained in water pumped from a domestic supply submersible pump than from a pump operating from a drill rig immediately following well development. Drill rig pumps are primarily used to develop wells after installation. They are designed to pump at very high flow rates specifically to remove fine sediment from the well bore and in proximity to the well screen. Our results indicate that, instead of field‐filtering, collecting initial samples from household plumbing can also reduce variability between driller‐collected regulatory samples and samples collected months after well construction.

Homeowners only use water sourced from plumbing, so it could benefit homeowners if the protocols for initial regulatory sampling were specific enough to yield consistent samples for representative measurement of As concentrations in drinking water collected from plumbing. Collection of regulatory samples from plumbing rather than from the rig on the day of well construction for determination of As concentration to comply with well construction code for potable water supply can be communicated to well‐drilling contractors, homeowners, and others, and could be implemented.

#### Sample Collection Timing

**Figure 8:**
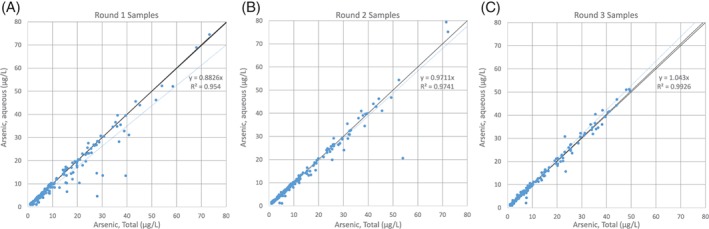
Total vs. aqueous arsenic concentrations over time. Over time the data (blue) approach the 1:1 line (black) and the degree of similarity increases as measured by R ^2^. μg/L, micrograms per liter.

Measured As concentrations are less variable when samples are collected months after the well is drilled. Aqueous and total As concentrations become more similar over time, as illustrated in Figures 7 and 8. Figure 8 illustrates that over time the total and aqueous As concentrations become very similar to one another, plotting very nearly along the 1:1 line. Most of the PPW results also indicate that total and aqueous As concentration samples collected during Rounds 2 and 3 are not different from one another. Conversely, the PPW results indicate that Round 1 total and aqueous concentrations are different. Study results are consistent with the finding of Erickson (2005), who compared total and aqueous As concentrations in new wells and long‐established wells. Aqueous and total As sample results were more similar from long‐established wells, and were less similar from newly drilled wells when water samples were collected from the drill rig. It is reasonable to expect that less solid material will be entrained in pumped water after a well has been established and operating for years.

Collecting As compliance samples at least 6 months after well construction can be communicated to well drillers and is possible to implement, but a second trip to the well and coordination with homeowners would be necessary. More importantly, waiting 6 months would mean either a delay putting the well into service or possibly exposing the new well owners to elevated concentrations of As in their drinking water for a period of time.

Finally, our study does not support the conclusion (anecdotally‐derived message) that As concentrations in well water decrease over time. For example, of the 8 Northeast region bedrock wells with waters having initial elevated As concentrations, 2 wells showed a decrease in As concentration over time, but 2 wells had a concentration increase. Arsenic concentrations in 3 of 8 wells were stable. One well had an As concentration decrease of about 50% yet continued to have a concentration of concern, above 10 μg/L. Figure 6 illustrates the direction of As concentration change (increase or decrease) and the magnitude of increase or decrease over time for all study wells. The percentage of wells that have waters with As concentration increases is about the same as those with decreases. However, the percentage of wells with large As concentration increases (more than 10 μg/L increase) is higher than the percentage of wells with a large As concentration decrease.

## Conclusions

This study examines the effect of several sample collection protocols on measured arsenic concentrations in newly‐constructed domestic drinking water wells in three regions of Minnesota, USA, where elevated arsenic concentrations in drinking water are common. Arsenic concentration measurements from repeated sampling at wells were less variable when (1) samples were filtered, (2) samples were collected from household plumbing rather than from a drill rig pump, and (3) samples were collected several months after well construction. Arsenic concentration changes over time are about equally likely to increase as to decrease. Results support the hypothesis that particulates and fine aquifer sediments entrained in arsenic samples or other artifacts of drilling disturbance cause variability in arsenic concentrations in well water.

Regulatory agencies could adopt improved water sampling guidance or rules that include field filtration, sampling collection point, or sample collection timing requirements. These results can inform efforts by regulatory and public health agencies to communicate with well drillers and homeowners about factors that affect water quality at wells and at plumbing fixtures, and to support the value of periodic re‐sampling for private well owners. Establishing specific protocols for collection of water quality samples from new drinking water wells could improve the utility of sampling and analysis following well drilling and development for potable supply. Homeowners only use water sourced from plumbing, so it could benefit homeowners if the initial regulatory sampling offered are representative measurement of As concentrations in drinking water collected from plumbing.

Reducing variability in water quality reporting also benefits secondary uses of water quality data, for example in water quality modeling. Highly variable measured arsenic concentrations weaken models by increasing categorical misclassifications or introducing uncertainty into continuous concentration variables. As water quality modeling and other interpretive activities that rely on existing water quality and other data expand (Yang et al. 2014; Nolan et al. 2015; Ayotte et al. 2016; Rosencrans et al. 2016; Tesoriero et al. 2017), dependence upon reliable data quality becomes ever more important.

This study also demonstrates that simple distribution statistics (for example, box plots) are not adequate to discern concentration differences in water quality samples influenced by the collection point, techniques, or timing at a single location. Pair‐wise comparisons using appropriate non‐parametric statistical methods are necessary to examine which factors influence or bias arsenic measurements at individual locations. Descriptive statistics (such as averages) that look at distributions of arsenic concentrations over time and space are adequate and reasonable for describing regional occurrence. But individual well owners do not drink water with an average arsenic concentration. Therefore, it is important to understand and evaluate the factors that bias initial arsenic measurements in an individual new well toward being different from the actual longer‐term arsenic concentration in that well.

## Authors' Note

The author(s) does not have any conflicts of interest or financial disclosures to report.

## Supporting information


**Table S1.** Summary of the standard protocols used by the Minnesota Department of Health for water sample collection, processing, and analysis
**Table S2.** Summary of Relative Percent Differences (RPD) between 75 reference (Ref) and replicate (Rep) samples collected by the Minnesota Department of Health
**Table S3.** Summary results from the Paired Prentice Wilcoxon (PPW) test comparing reference to replicate samples' distributions
**Table S4.** Summary of Minnesota Department of Health lab results from field blanks
**Table S5.** Summary of samples collected and collection lag times
**Table S6.** Detailed summary of Paired Prentice Wilcoxon test results p‐values, by region
**Table S7.** Arsenic above/below 10 μg/L category swap summary
