# Supplementary material for: How or When Samples Are Collected Affects Measured Arsenic Concentration in New Drinking Water Wells
Source: Ground Water. 2018 Mar 6;56(6):921–33. doi: 10.1111/gwat.12643 (PMC6282803; doi:10.1111/gwat.12643)
Supplement: Supplementary file 1 — Table S1. Summary of the standard protocols used by the Minnesota Department of Health for water sample collection, processing, and analysis Table S2. Summary of Relative Percent Differences (RPD) between 75 reference (Ref) and replicate (Rep) samples collected by the Minnesota Department of Health Table S3. Summary results from the Paired Prentice Wilcoxon (PPW) test comparing reference to replicate samples' distributions Table S4. Summary of Minnesota Department of Health lab results from field blanks Table S5. Summary of samples collected and collection lag times Table S6. Detailed summary of Paired Prentice Wilcoxon test results p‐values, by region Table S7. Arsenic above/below 10 μg/L category swap summary [file GWAT-56-921-s001.docx]

**SUPPORTING INFORMATION for “How or when samples are collected affects measured arsenic concentration in new drinking water wells”**

Melinda L. Erickson, corresponding author

US Geological Survey, merickso@usgs.gov

Helen F. Malenda,

Colorado School of Mines

Emily C. Berquist,

Minnesota Department of Health

**Table SI 1:** Summary of the standard protocols used by the Minnesota Department of Health for water sample collection, processing, and analysis.

| Analysis | Filtering | Bottle | Preservative | Method | Citation |
| --- | --- | --- | --- | --- | --- |
| Total Arsenic | None | 250 mL HDPE bottle | 2.50 mL 20% HNO_3_, refrigeration | EPA 200.8 | EPA. 1994. “Method 200.8: Determination of Trace Elements in Waters and Wastes by Inductively Coupled Plasma-Mass Spectrometry,” Revision 5.4 |
| Aqueous Arsenic | 0.45 micron pore size filter, container or disk | 250 mL HDPE bottle | 2.50 mL 20% HNO_3_, refrigeration | EPA 200.8 | EPA. 1994. “Method 200.8: Determination of Trace Elements in Waters and Wastes by Inductively Coupled Plasma-Mass Spectrometry,” Revision 5.4 |

**Table SI 2:** Summary of Relative Percent Differences (RPD) between 75 reference (Ref) and replicate (Rep) samples collected by the Minnesota Department of Health. TAs, Total arsenic; AqAs, Aqueous (dissolved) arsenic; ND, not detected.

| **Date** | **Well** | **TAs_Ref** | **TAs_Rep** | **RPD** | **AqAs_Ref** | **AqAs_Rep** | **RPD** |
| --- | --- | --- | --- | --- | --- | --- | --- |
| **7/16/2014** | **805809** | 1410 | 1630 | **14%** | 1340 | 1330 | **1%** |
| **7/31/2014** | **804556** | 7.27 | 7.64 | **5%** | 7.11 | 7.13 | **0%** |
| **8/20/2014** | **801304** | 4.34 | 4.44 | **2%** | 4.81 | 4.73 | **2%** |
| **9/30/2014** | **802030** | 2.73 | 3.06 | **11%** | 2.48 | 2.53 | **2%** |
| **10/21/2014** | **805320** | 3.46 | 3.18 | **8%** | 3.78 | 3.75 | **1%** |
| **10/21/2014** | **807932** | 11.5 | 11.3 | **2%** | 12 | 11.9 | **1%** |
| **3/10/2015** | **810093** | 5.94 | 5.88 | **1%** | 5.14 | 5.06 | **2%** |
| **5/7/2015** | **810088** | 6.77 | 6.93 | **2%** | 7.4 | 7.53 | **2%** |
| **6/19/2015** | **793806** | ND | ND | **ND** | ND | ND | **ND** |
| **6/19/2015** | **810877** | 2.26 | 2.82 | **22%** | 2.07 | 2.16 | **4%** |
| **9/9/2014** | **806744** | ND | ND | **ND** | ND | ND | **ND** |
| **11/6/2014** | **809231** | 22.1 | 22.4 | **1%** | 18 | 18.5 | **3%** |
| **5/27/2015** | **807791** | 38.9 | 38.3 | **2%** | 32.8 | 32.8 | **0%** |
| **7/7/2015** | **810371** | 2.93 | 2.89 | **1%** | 2.95 | 2.9 | **2%** |
| **7/8/2015** | **811840** | 7.28 | 7.24 | **1%** | 7.1 | 7.28 | **3%** |
| **7/17/2015** | **808869** | 8.58 | 8.63 | **1%** | 9.43 | 8.99 | **5%** |
| **7/31/2015** | **809534** | ND | ND | **ND** | ND | ND | **ND** |
| **8/18/2015** | **799534** | 26.6 | 26.3 | **1%** | 26.9 | 26.9 | **0%** |
| **8/25/2015** | **807557** | 11.6 | 11.3 | **3%** | 12.4 | 12.4 | **0%** |
| **6/22/2016** | **809246** | 4.86 | 4.88 | **0%** | 5.87 | 5.83 | **1%** |
| **6/2/2015** | **810792** | 2.24 | 2.06 | **8%** | 2.48 | 2.14 | **15%** |
| **7/16/2015** | **808686** | 8.95 | 8.87 | **1%** | 8.95 | 9.05 | **1%** |
| **8/4/2015** | **802855** | 1.19 | 1.34 | **12%** | 1.34 | 1.13 | **17%** |
| **9/22/2015** | **813782** | 4.85 | 4.83 | **0%** | 4.98 | 5 | **0%** |
| **4/8/2015** | **804554** | 1.13 | 1.22 | **8%** | 1.08 | 1.05 | **3%** |
| **4/8/2015** | **804556** | 3.29 | 3.05 | **8%** | 3.82 | 3.8 | **1%** |
| **4/8/2015** | **804559** | 13 | 12.8 | **2%** | 13.4 | 13.2 | **2%** |
| **4/14/2015** | **804558** | 2.6 | 2.66 | **2%** | 2.32 | 2.29 | **1%** |
| **4/17/2015** | **802607** | 3.75 | 3.73 | **1%** | 3.86 | 3.91 | **1%** |
| **4/29/2015** | **802033** | ND | ND | **ND** | ND | ND | **ND** |
| **5/28/2015** | **808088** | 2.42 | 2.36 | **3%** | 2.47 | 2.39 | **3%** |
| **9/30/2015** | **808505** | 8.36 | 8.26 | **1%** | 9.02 | 8.52 | **6%** |
| **10/21/2015** | **807839** | 33 | 33.2 | **1%** | 32.7 | 32.4 | **1%** |
| **2/10/2015** | **799511** | 12.2 | 10.4 | **16%** | 9.96 | 10.3 | **3%** |
| **2/10/2015** | **806922** | ND | ND | **ND** | ND | ND | **ND** |
| **3/23/2015** | **802942** | 24.6 | 24.5 | **0%** | 23.4 | 23.4 | **0%** |
| **4/10/2015** | **804106** | ND | ND | **ND** | ND | ND | **ND** |
| **6/24/2015** | **809530** | 12.1 | 12.2 | **1%** | 11 | 10.8 | **2%** |
| **10/26/2015** | **808869** | 7.19 | 7.51 | **4%** | 7.39 | 7.25 | **2%** |
| **11/18/2015** | **812811** | 1.66 | 1.69 | **2%** | 1.82 | 1.72 | **6%** |
| **12/4/2015** | **807557** | 12.1 | 11.8 | **3%** | 12.7 | 13.2 | **4%** |
| **3/4/2016** | **809544** | 25.8 | 25.8 | **0%** | 26.5 | 26.2 | **1%** |
| **6/29/2016** | **812851** | ND | ND | **ND** | ND | ND | **ND** |
| **6/10/2016** | **810829** | ND | ND | **ND** | ND | ND | **ND** |
| **11/14/2015** | **808686** | 8.92 | 8.89 | **0%** | 9.57 | 9.83 | **3%** |
| **4/29/2016** | **802855** | ND | ND | **ND** | ND | ND | **ND** |
| **4/30/2016** | **811366** | 1.45 | 1.46 | **1%** | 1.53 | 1.53 | **0%** |
| **5/20/2016** | **810799** | 1.13 | ND | **ND** | ND | ND | **ND** |
| **5/20/2016** | **813094** | ND | ND | **ND** | ND | ND | **ND** |
| **5/20/2016** | **813782** | 4.95 | 4.85 | **2%** | 5.03 | 4.97 | **1%** |
| **10/2/2015** | **798297** | 21.9 | 21.8 | **0%** | 22.8 | 23 | **1%** |
| **4/14/2016** | **805317** | 2.38 | 2.48 | **4%** | 2.56 | 2.56 | **0%** |
| **4/14/2016** | **805320** | 4.38 | 4.33 | **1%** | 4.38 | 4.51 | **3%** |
| **5/19/2016** | **799914** | 2.33 | 2.27 | **3%** | 2.39 | 2.38 | **0%** |
| **6/10/2016** | **804308** | 1.17 | 1.14 | **3%** | 1.43 | 1.37 | **4%** |
| **6/29/2016** | **810084** | 4.56 | 4.48 | **2%** | 5.19 | 4.91 | **6%** |
| **7/8/2016** | **812126** | ND | ND | **ND** | ND | ND | **ND** |
| **7/9/2016** | **810877** | 1.95 | 1.96 | **1%** | 1.88 | 1.73 | **8%** |
| **7/13/2016** | **804640** | 15.6 | 15.4 | **1%** | 15.9 | 15.9 | **0%** |
| **7/20/2016** | **812131** | ND | ND | **ND** | ND | ND | **ND** |
| **9/13/2016** | **808865** | 5.33 | 5.8 | **8%** | 6.58 | 6.43 | **2%** |
| **9/30/2015** | **807608** | 5.37 | 6.03 | **12%** | 6.79 | 6.84 | **1%** |
| **10/29/2015** | **799524** | 23.5 | 23.8 | **1%** | 15.7 | 20.9 | **28%** |
| **10/29/2015** | **802947** | 21.4 | 21.3 | **0%** | 20 | 20 | **0%** |
| **5/28/2016** | **811831** | 9.41 | 9.43 | **0%** | 9.62 | 9.48 | **1%** |
| **5/31/2016** | **810903** | 35.8 | 35 | **2%** | 34 | 33.5 | **1%** |
| **7/6/2016** | **807791** | 36.6 | 36.5 | **0%** | 34.6 | 36.1 | **4%** |
| **7/25/2016** | **810370** | 1.18 | 1.18 | **0%** | 1.17 | 1.22 | **4%** |
| **8/10/2016** | **809451** | 1.17 | 1.12 | **4%** | 1.2 | 1.18 | **2%** |
| **8/26/2016** | **812776** | 22.4 | 22.2 | **1%** | 20.2 | 21.4 | **6%** |
| **9/7/2016** | **814781** | 20.2 | 19.9 | **1%** | 21.4 | 20.7 | **3%** |
| **7/8/2016** | **810776** | 1.23 | 1.18 | **4%** | 1.3 | 1.28 | **2%** |
| **7/9/2016** | **808686** | 9.8 | 9.44 | **4%** | 9.4 | 9.41 | **0%** |
| **7/21/2016** | **807210** | 8.02 | 6.75 | **17%** | 7.94 | 7.95 | **0%** |
| **8/5/2016** | **812105** | 9.43 | 9.34 | **1%** | 9.54 | 9.34 | **2%** |
| **Maximum** |  |  |  | **22%** |  |  | **28%** |
| **Average** |  |  |  | **4%** |  |  | **3%** |

**Table SI 3:** Summary results from the Paired Prentice Wilcoxon (PPW) test comparing reference to replicate samples’ distributions. PPW Z, PPW Z-statistic; n, number of samples; p-value, significance factor; h_o_, null hypothesis that distributions are the same.

| **Reference versus replicate data** | | | | |
| --- | --- | --- | --- | --- |
| **Analyte** | **PPW Z** | **n** | **p-value** | **h_o_** |
| Total Arsenic | 1.0808 | 75 | 0.2798 | accepted |
| Aqueous Arsenic | 1.5035 | 75 | 0.1327 | accepted |

**Table SI 4:**  Summary of Minnesota Department of Health lab results from field blanks. [TAs], concentration of total arsenic; RL, reporting level; [AqAs], concentration of aqueous arsenic; DI, deionized; µg/L, micrograms per liter.

| **Collection Date** | **Sample ID** | **Sampling Point** | **[TAs]** | **RL** | **[AqAs]** | **RL** |
| --- | --- | --- | --- | --- | --- | --- |
| 4/15/2015 | 15D1139-01 | Peristaltic Pump- DI water | ND | 1 µg/l | ND | 1 µg/l |
| 4/15/2015 | 15D1145-01 | Syringe Check- DI water | ND | 1 µg/l | ND | 1 µg/l |
| 4/23/2015 | 15E0345-01 | Peristaltic Pump- DI water | ND | 1 µg/l | ND | 1 µg/l |
| 4/23/2015 | 15E0351-01 | Syringe Check- DI water | ND | 1 µg/l | ND | 1 µg/l |
| 5/1/2015 | 15E0030-01 | Peristaltic Pump- DI water | ND | 1 µg/l | ND | 1 µg/l |
| 5/1/2015 | 15E0026-01 | Syringe Check- DI water | ND | 1 µg/l | ND | 1 µg/l |
| 6/3/2016 | 16F1483-01 | Peristaltic Pump- DI water | ND | 1 µg/l | ND | 1 µg/l |
| 6/3/2016 | 16F1482-01 | Syringe Check- DI water | ND | 1 µg/l | ND | 1 µg/l |
| 9/24/2016 | 16J0011-01 | Hand Pump- DI water | ND | 1 µg/l | ND | 1 µg/l |
| 9/29/2016 | 16J0012-01 | Peristaltic Pump- DI water | ND | 1 µg/l | ND | 1 µg/l |

**Table SI 5:** Summary of samples collected and collection lag times

| **Region** | **Round 1** | | **Round 2** | | **Round 3** | | **Study** |
| --- | --- | --- | --- | --- | --- | --- | --- |
|  | Count | First sample collection date (earliest, median, latest) | Count | Lag time from Round 1  Mean (min, med, max) days,  Mean (min, med, max) months | Count | Lag time from Round 2  Mean (min, med, max) days, Mean (min, med, max) months | Lag time, Round 1 to Round 3  Mean (min, med, max) days,  Mean (min, med, max) months |
| All | 254 | 5/14/2014 3/17/15 (median) 10/1/2015 | 251 | 173 (58, 155, 396) 5.2 (1.0, 5.0, 13.0) | 245 | 262 (58, 261, 455) 8.1 (1.0, 8.0, 14.0) | 435 (319, 398, 721) 13.8 (10.0, 13.0, 23.0) |
| Northeast | 49 | 6/11/2014 7/16/15 (median) 9/30/2015 | 46 | 169 (84, 155, 279) 5.1 (2.0, 5.0, 9.0) | 44 | 221 (99, 224, 351) 6.8 (3.0, 7.0, 11.0) | 390 (319, 380, 527) 12.3 (10.0, 12.0, 17.0) |
| Northwest | 101 | 5/14/2014 6/4/15 (median) 10/1/2015 | 101 | 161 (58, 136, 396) 4.8 (1.0, 4.0, 13.0) | 100 | 229 (58, 251, 343) 7.1 (1.0, 8.0, 11.0) | 391 (348, 386, 715) 12.4 (11.0, 12.0, 23.0) |
| Central | 104 | 5/21/2014 10/9/2014 (median) 9/1/2015 | 104 | 187 (96, 182, 344) 5.7 (3.0, 6.0, 11.0) | 101 | 310 (123, 324, 455) 9.7 (4.0, 10.0, 14.0) | 498 (349, 469, 721) 15.8 (11.0, 15.0, 23.0) |

**Table SI6:** Detailed summary of Paired Prentice Wilcoxon test results p-values, by region. Interpretation of p-values provided in Table 1. Bold indicated comparisons that are not different from one another; null hypothesis accepted.
[Mo, month; TAs, total arsenic; AqAs, aqueous arsenic; MDH, Minnesota Department of Health.]

**Table SI-7:** Arsenic above/below 10 µg/L category swap summary

[TAs, total arsenic; AqAs, aqueous arsenic; µg/L, micrograms per liter; MDH, Minnesota Department of Health]

| Comparison of category changes | 0-Month samples compared to 3-6 Month AqAs | | | | 0-Month samples compared to 12 Month AqAs | | | | 3-6 Month AqAs compared to 12 Month AqAs | |
| --- | --- | --- | --- | --- | --- | --- | --- | --- | --- | --- |
|  | ≥ 10 to <10 µg/L | | <10 to ≥ 10 µg/L | | ≥ 10 to <10 µg/L | | <10 to ≥ 10 µg/L | |  |  |
|  | Driller TAs | MDH AqAs | Driller TAs | MDH AqAs | Driller TAs | MDH AqAs | Driller TAs | MDH AqAs | >10 to <10 | <10 to >10 |
| Number of sample pairs | 248 | 251 | 248 | 251 | 241 | 244 | 241 | 244 | 242 | 242 |
| Number of category changes | 21 | 7 | 8 | 9 | 21 | 6 | 9 | 9 | 2 | -2 |
| Percent category changes | 8.5% | 2.8% | 3.2% | 3.6% | 8.8% | 2.5% | 3.7% | 3.7% | 0.8% | 0.8% |
